# Comparison of 2D, 2.5D, and 3D segmentation networks for mandibular canals in CBCT images: a study on public and external datasets

**DOI:** 10.1186/s12903-025-06483-4

**Published:** 2025-07-08

**Authors:** Su Yang, Jong Soo Jeong, Dahyun Song, Ji Yong Han, Sang-Heon Lim, Sujeong Kim, Ji-Yong Yoo, Jun-Min Kim, Jo-Eun Kim, Kyung-Hoe Huh, Sam-Sun Lee, Min-Suk Heo, Won-Jin Yi

**Affiliations:** 1 Department of Applied Bioengineering, Graduate School of Convergence Science and Technology, Seoul National University, Seoul, 08826 South Korea; 2 Department of Dentistry, School of Dentistry, Seoul National University, Seoul, 08826 South Korea; 3 Interdisciplinary Program in Bioengineering, Graduate School of Engineering, Seoul National University, Seoul, 08826 South Korea; 4 Department of Electronics and Information Engineering, Hansung University, Seoul, 02876 South Korea; 5 Department of Oral and Maxillofacial Radiology and Dental Research Institute, School of Dentistry, Seoul National University, Seoul, 03080 South Korea

**Keywords:** Deep learning, CBCT image, Mandibular canal, Image segmentation, 3D segmentation network

## Abstract

The purpose of this study was to compare the performances of 2D, 2.5D, and 3D CNN-based segmentation networks, along with a 3D vision transformer-based segmentation network, for segmenting mandibular canals (MCs) on the public and external CBCT datasets under the same GPU memory capacity. We also performed ablation studies for an image-cropping (IC) technique and segmentation loss functions. 3D-UNet showed the highest segmentation performance for the MC than those of 2D and 2.5D segmentation networks on public test datasets, achieving 0.569 ± 0.107, 0.719 ± 0.092, 0.664 ± 0.131, and 0.812 ± 0.095 in terms of JI, DSC, PR, and RC, respectively. On the external test dataset, 3D-UNet achieved 0.564 ± 0.092, 0.716 ± 0.081, 0.812 ± 0.087, and 0.652 ± 0.103 in terms of JI, DSC, PR, and RC, respectively. The IC technique and multi-planar Dice loss improved the boundary details and structural connectivity of the MC from the mental foramen to the mandibular foramen. The 3D-UNet demonstrated superior segmentation performance for the MC by learning 3D volumetric context information for the entire MC in the CBCT volume.

**Supplementary Information:**

The online version contains supplementary material available at 10.1186/s12903-025-06483-4.

## Introduction

The mandibular canal (MC) within the mandible is a tubular anatomical structure that begins at the mandibular foramen on the medial surface of the ascending mandibular ramus [1, 2]. Damage to the MC can lead to significant complications, including hypoesthesia, paresthesia, and dysesthesia, which affect speech, chewing, and overall quality of life [3, 4]. Therefore, accurate identification of the MC is essential in planning maxillofacial surgeries, such as dental implant placement and third molar extractions, to prevent inadvertent injury to the inferior alveolar nerve [4–6]. Cone-beam computed tomography (CBCT) is widely used in dentistry for 3D diagnosis and treatment planning due to its low radiation exposure, high spatial resolution, and cost-effective image acquisition [7, 8]. In pre-operative surgical planning, dental clinicians manually analyze the location and trajectory of the MC in CBCT images, which provide precise 3D localization and visualization of the MC within the mandible [9, 10]. However, manual segmentation of the MC in CBCT images by dental clinicians is labor-intensive, time-consuming, and subject to inter- and intra-observer variability [11]. Furthermore, the ambiguous cortical bone layer surrounding the MC and unclear medulla patterns make it challenging to identify the entire MC in low-contrast CBCT images [11, 12]. Therefore, an automatic method for segmenting the MC in CBCT images is needed to reduce the workload of dental clinicians.

As deep learning technologies have advanced, several studies have focused on segmenting anatomical structures in 3D medical data using convolutional neural network (CNN) and vision transformer (ViT) models [13–16]. Several CNN- and ViT-based networks have been proposed to automatically segment the MC in CBCT images [11, 17–19]. 2D CNNs are widely used to segment anatomical structures in medical images [20]. The most straightforward approach involves converting volume data into 2D image sequences along orthogonal planes and training a 2D CNN with 2D image inputs. With this method, it is difficult to capture the volumetric relationships between adjacent slices, which are crucial for accurate anatomical segmentation. To address that limitation, 3D CNNs have been introduced to capture and use 3D volumetric information about anatomical structures [20]. However, 3D CNNs require significantly more memory and computational resources than 2D CNNs. To balance the advantages of 2D and 3D segmentation networks, 2.5D segmentation networks have been proposed [20]. These networks separately train 2D CNNs on the axial, sagittal, and coronal planes and then combine the segmentation results from each 2D CNN using ensemble methods such as unanimous, affirmative, or majority voting approaches [20]. The 2.5D approach aims to leverage volumetric information across different planes while mitigating the memory demands and computational resources required by 3D CNNs.

Recently, ViT models have been adapted as a powerful alternative to CNNs for medical image segmentation due to their ability to capture long-range dependencies and contextual information more effectively than traditional CNNs [16]. However, ViT models require significantly more computational resources and memory than CNNs, which can be a limiting factor in real-world settings with restricted computational capabilities. Additionally, ViT models often need large training datasets to achieve optimal performance, which can be challenging in medical fields due to privacy concerns and the cost of data annotation.

Despite the various strengths of different deep learning models for the segmentation of anatomical structures in medical imaging, there is still significant debate about which training approaches, such as 2D, 2.5D, and 3D methods, are optimal for specific tasks [20, 21]. As far as we know, no previous studies have compared the MC segmentation performances of 2D, 2.5D, and 3D networks on CBCT images. Therefore, the purpose of this study was to compare the segmentation performances of the 2D, 2.5D, and 3D CNN-based networks as well as the 3D ViT-based network for the MC in the public and external CBCT datasets under the same GPU memory capacity. We also performed ablation studies for an image-cropping (IC) technique and segmentation loss functions [11]. We trained the 2D, 2.5D, and 3D networks using only the public training dataset and evaluated their segmentation performance on the public and external test datasets.

## Materials and Methods

### Data acquisition and preparation

**Fig. 1:**
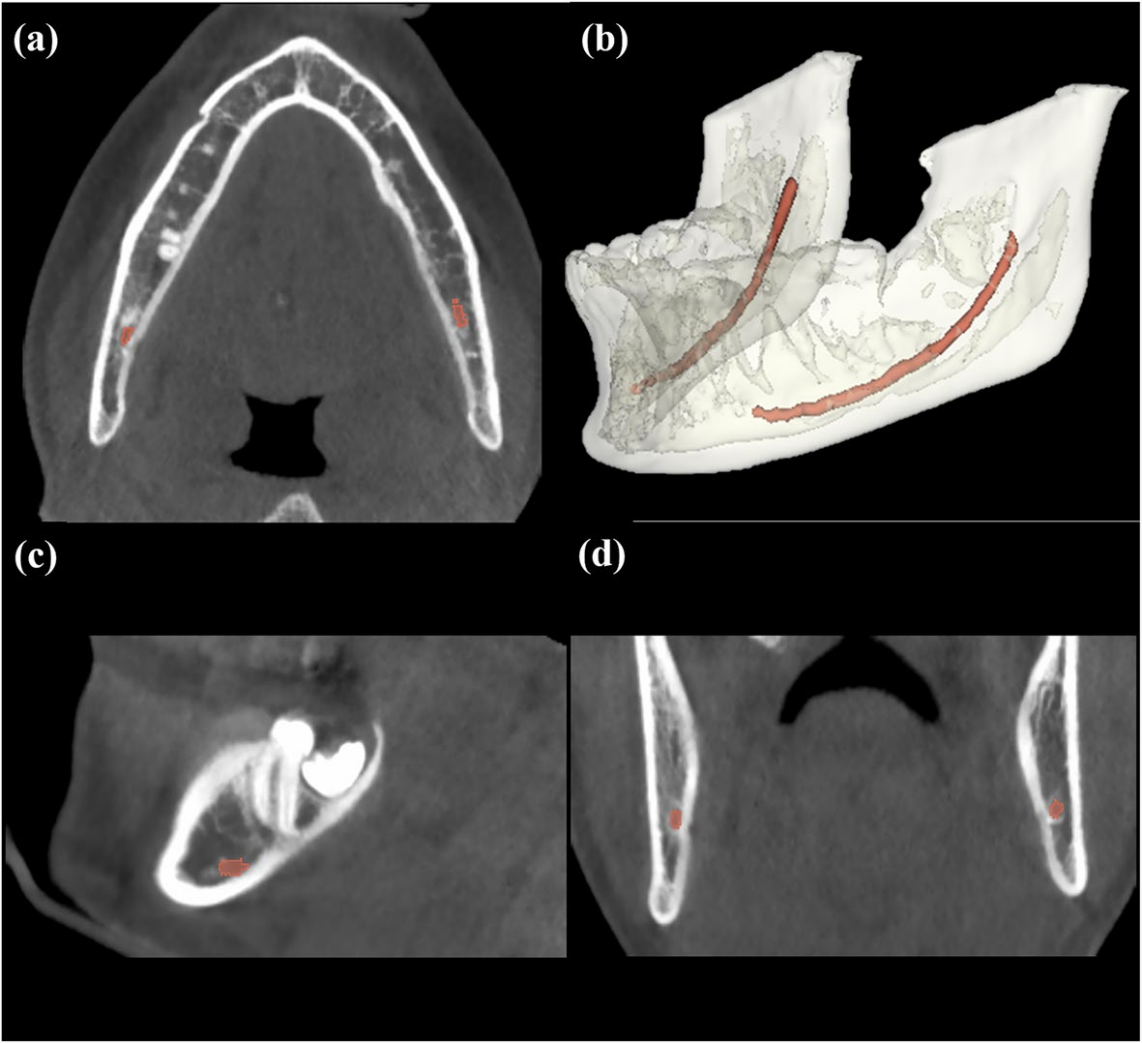
CBCT images with the ground truth of the mandibular canals in red. **a**, **c** and **d** show axial, sagittal, and coronal CBCT images, respectively. **b** shows a 3D reconstruction of the mandible (white) and mandibular canals (red)

We used the publicly accessible ToothFairy2023 challenge dataset hosted by the Medical Image Computing and Computer-Assisted Intervention Society [22, 23]. The dataset includes 153 CBCT volumes obtained under the condition of 110 kVp and 3 mA using a CBCT modality (NewTom, QR, Verona, Italy) from the Affidea center located in Modena, Italy. The CBCT volumes have dimensions ranging from 148 × 265 × 312 to 178 × 423 × 463 with voxel sizes of 0.3 × 0.3 × 0.3mm^3^. The 153 CBCT volumes were randomly split into 91, 31, and 31 volumes for the public training, validation, and test datasets, respectively. To ensure a fair comparison of 2D, 2.5D, and 3D segmentation networks while adhering to memory constraints, all the CBCT volumes were resized to 128 × 256 × 256 pixels. The ground truth of the ToothFairy2023 challenge dataset was manually annotated by medical professionals with over five years of experience in the maxillofacial field [22, 23] (Fig. 1). The ToothFairy2023 challenge dataset is accessible to the public after user registration and can be downloaded from https://ditto.ing.unimore.it/toothfairy/.

To evaluate the segmentation performance on the external test dataset, we collected 30 CBCT volumes from 30 patients who underwent dental implant surgeries at Seoul National University Dental Hospital (2020–2021). These CBCT images were obtained at 75 to 120 kVp and 7 to 10 mA using CBCT (DENTRI, HDX WILL Corp, Seoul, South Korea). These images have dimensions of 291 × 841 × 841 pixels, voxel sizes of 0.3 × 0.3 × 0.3 mm^3^, and 16-bit depth. The ground truth of the external test dataset was manually annotated by an oral and maxillofacial radiologist with over five years of experience using software (3D Slicer for Windows 10, Version 4.10.2; MIT, Massachusetts, USA). This study was performed with approval from the institutional review board of Seoul National University Dental Hospital (ERI18001) and was conducted in accordance with the Declaration of Helsinki. The institutional review board of Seoul National University Dental Hospital approved the waiver for informed consent because this was a retrospective study.

In this study, we compare the performance of the segmentation networks with and without the IC technique [11]. Typically, the MC exists symmetrically on the left and right sides, allowing each side to be used as a separate training dataset. For this purpose, the CBCT volume and corresponding ground truth were bisected along the sagittal plane. Then, the right side was horizontally flipped to the left side to ensure uniformity in orientation, resulting in cropped CBCT datasets with dimensions of 128 × 256 × 128. All CBCT volumes were normalized using min–max normalization, and the pixel intensity was scaled to a range of 0.0 to 1.0. The segmentation networks take CBCT images as input and then directly output segmentation results of the mandibular canals.

### 2D segmentation networks

**Fig. 2:**
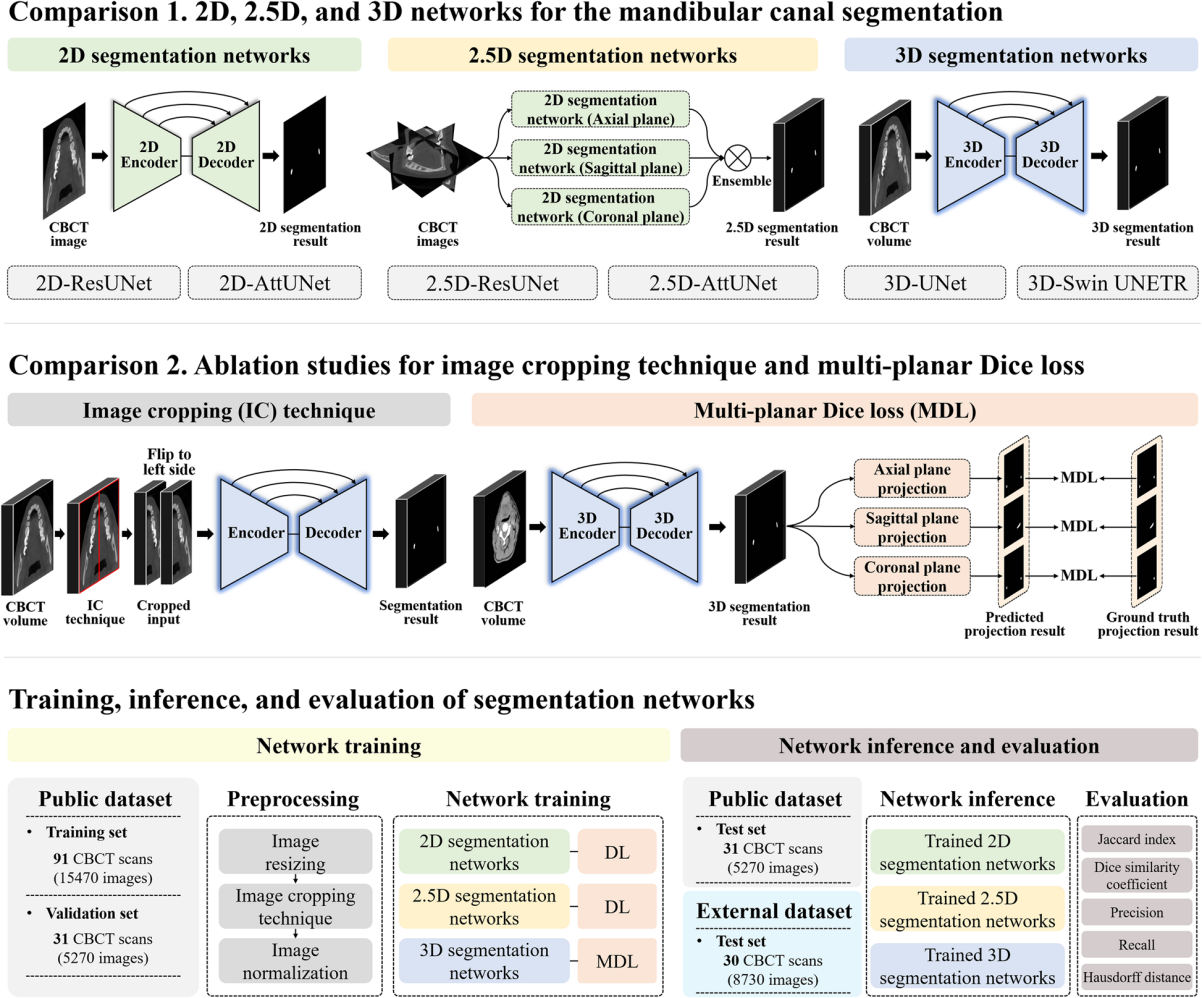
Overview illustrations of the 2D, 2.5D, and 3D segmentation networks and their training, inference, and evaluation processes with the size of the public and external datasets

In this study, we used 2D U-Net with a ResNet101 backbone (2D-ResUNet) [24] and 2D Attention U-Net (2D-AttUNet) [25] as our 2D segmentation networks (Fig. 2). 2D-ResUNet has a U-shaped architecture consisting of a ResNet101 backbone [24] and a decoder [26]. The encoder comprises several convolutional layers and pooling operations that progressively reduce the spatial dimensions while extracting increasingly abstract features. The decoder involves up-sampling operations and convolutional layers, which gradually restore the original image resolution while combining the features extracted by the encoder. Skip connections are used between corresponding layers of the encoder and decoder to ensure that spatial information is preserved and used effectively during the up-sampling process. This structure allows the network to perform precise localization and segmentation tasks by leveraging both global context and fine-grained details. 2D-AttUNet enhances the standard U-Net by incorporating an additive attention mechanism into the skip connections [25]. These attention gates help the network focus on important features and ignore less relevant information, improving the network's ability to segment structures accurately against complex backgrounds. This attention mechanism allows the segmentation network to achieve more accurate and robust segmentation results than the traditional U-Net.

### 2.5D segmentation networks

For the 2.5D segmentation approach, we trained separate 2D segmentation networks using 2D image inputs from the axial, sagittal, and coronal planes (Fig. 2). The 2.5D segmentation networks took a stack of continuous 2D prediction results from those three 2D segmentation networks [20]. Then, the ensemble approaches using unanimous, affirmative, and majority methods were applied to the 2.5D segmentation networks to improve predictive performance by combining the prediction results from the axial, coronal, and sagittal planes [20]. The unanimous method considers the final ensemble result to be true if the prediction results in all three planes are true. The affirmative method considers the final ensemble result to be true if the prediction result in any one of the three planes is true. The majority method considers the final ensemble result to be true if the prediction results in the majority of the three planes are true.

### 3D segmentation networks

We employed 3D segmentation networks (3D-UNet and 3D-Swin UNETR) to effectively capture volumetric information in the CBCT volumes [27, 28] (Fig. 2). 3D-UNet has a U-shaped architecture that is similar to that of 2D-UNet and consists of a 3D encoder and a 3D decoder [27]. In 3D-UNet, we used 3D convolutional blocks, max-pooling operations, and 3D transposed convolutional blocks. The architecture followed a specific channel progression: starting from the input channels, the number of channels increased sequentially to 8, 16, 32, and 64 in the 3D encoder and then decreased symmetrically to 32, 16, and 8 in the 3D decoder before the output layer. The output layer consisted of a 3 × 3 × 3 convolutional layer and the Softmax activation function.

We also adopted 3D-Swin UNETR, which is based on a ViT approach, for 3D segmentation of the MC in CBCT volumes [28]. 3D-Swin UNETR has a U-shaped Swin transformer encoder and convolutional decoder. The Swin transformer encoder uses a hierarchical transformer with shift windows and self-attention to capture long-range dependencies and volumetric information in CBCT volumes. The features from the Swin transformer encoder are connected to the convolutional decoder at each layer using skip connections. The convolutional decoder consists of 3D residual blocks and 3D deconvolutional layers. In 3D-Swin UNETR, the embedding dimension, feature size, and window size were set as 768, 48, and 7, respectively. A comparative overview of 2D, 2.5D, and 3D training approaches in deep learning is given in Table 1.

**Table 1:** A comparative overview of 2D, 2.5D, and 3D training approaches in deep learning

Training approaches	Advantages	Disadvantages	Key differences
2D approach	✓ Widely used in deep learning applications ✓ Low computational cost ✓ Fast training and inference time	✓ Lack of spatial continuity of inter-slices ✓ Limited ability to leverage 3D contextual information	✓ Processes each 2D slice independently without considering inter-slice relationships
2.5D approach	✓ Utilizes multiple plane information (axial, sagittal, and coronal) ✓ Enhanced inter-slice relationships compared to a 2D approach	✓ Requires an ensemble-based combination of multiple planes ✓ Still lacks 3D contextual information compared to a 3D training approach	✓ Combines segmentation results from 2D axial, sagittal, and coronal planes to incorporate 3D information
3D approach	✓ Captures 3D contextual information ✓ Consider inter-slice relationships in network training	✓ High computational and memory requirements ✓ More extended training and inference time ✓ Requires more extensive labeled datasets	✓ Processes the entire 3D volume ✓ Learning volumetric relationships across the CBCT images

### Segmentation loss functions

For network training, we used a Dice similarity coefficient loss (*DL*) that is widely adopted in image segmentation tasks to measure the similarity between the ground truth and predicted results [11]. The *DL* ranges from 0, indicating no overlap, to 1, indicating perfect overlap, and is defined as:

1 $$DL\left(x,y\right)=\frac{(2{\sum }_{i}^{n}{x}_{i}{y}_{i})+\epsilon }{\left({\sum }_{i}^{n}{x}_{i}+{\sum }_{i}^{n}{y}_{i}\right)+\epsilon },$$

where $n$, $x$, and y are the number of pixels, the predicted result, and the ground truth, respectively. $\epsilon$ is a constant value to prevent division by zero and set as 10^–3^.

To improve the structural continuity of the MC volume, we adopted the multi-planar Dice similarity coefficient loss ($MDL$) in the 3D segmentation networks [11]. MDL is calculated from the multi-planar 2D Dice losses by projecting the MC volume predicted by the 3D segmentation network onto each 2D plane and the ground truth of the MC volume. The MDL is defined as:

2 $$MDL\left(x,y\right)=aDL\left(x,y\right)+sDL\left(x,y\right)+cDL(x,y)$$

where $aDL$, $sDL,$ and $cDL$ are $DL$ s calculated on the axial, sagittal, and coronal planes, respectively. The $MDL$ is calculated as the sum of the $aDL$, $sDL$, and $cDL$ scores.

### Training details

To ensure a fair comparison between the segmentation networks, all models were trained under consistent conditions. We used the Adam optimizer for all training processes with a learning rate of 10^–3^. The segmentation networks were trained during 200 epochs. The batch size was set to five for the 2D and 2.5D segmentation networks, whereas for the 3D segmentation networks, a batch size of 1 was used due to computational memory constraints. All segmentation networks were implemented using PyTorch on an NVIDIA RTX A6000 GPU.

### Performance evaluation metrics

We evaluated the performance of the 2D, 2.5D, and 3D segmentation networks using the Jaccard index ($JI=\frac{TP}{TP+FN+FP}$), Dice similarity coefficient ($DSC=\frac{2TP}{2TP+FN+FP}$), precision ($PR=\frac{TP}{TP+FP}$), and recall ($RC=\frac{TP}{TP+FN}$) [29]. *TP*, *FP*, and *FN* represent true positive, false positive, and false negative between the ground truth and segmentation results from segmentation networks, respectively. Higher JI, DSC, PR, and RC values indicate better MC segmentation performance.

A statistical analysis of variance test was conducted to evaluate the segmentation performance across 2D, 2.5D, and 3D segmentation networks using SPSS Statistics for Windows 11 (Version 29.0; IBM, Armonk, New York, USA), with a statistical significance threshold (*p*-value) set at 0.05.

## Results

The segmentation performances of the 2D, 2.5D, and 3D networks were evaluated on public and external test datasets not used for network training. Furthermore, we performed ablation studies for an IC technique and segmentation loss functions. On the public test dataset, 3D-UNet showed the highest segmentation performance, better than the 2D and 2.5D segmentation networks (Table 2). 3D-UNet achieved 0.569 ± 0.107, 0.719 ± 0.092, and 0.812 ± 0.095 in terms of JI, DSC, and RC, respectively. Significant differences were observed in the PR between 3D-UNet and the other segmentation networks including 2D-ResUNet and 2.5D-ResUNet (*p*-value < 0.05). 2.5D-AttUNet showed the best PR value of 0.715 ± 0.120 and outperformed the 2D segmentation networks in terms of JI, DSC, and PR (Table 2). 2.5D-AttUNet with the majority ensemble method showed better segmentation performance than 2.5D-ResUNet with the majority ensemble method. The segmentation performance in terms of JI, DSC, PR, and RC by 2.5D segmentation networks using different ensemble methods (unanimous, affirmative, and majority) is shown in Supplementary Table S1. The segmentation performance of the 2D segmentation networks trained for the axial, sagittal, and coronal planes is shown in Supplementary Table S2.

**Table 2:** Segmentation performances in terms of the Jaccard index (JI), Dice similarity coefficient (DSC), precision (PR), and recall (RC) for the mandibular canals from 2D, 2.5D, and 3D segmentation networks on the public test dataset

Segmentation networks	JI	DSC	PR	RC
2D-ResUNet	0.518 ± 0.109	0.675 ± 0.097	0.676 ± 0.122^*^	0.699 ± 0.127
2D-AttUNet	0.521 ± 0.123	0.676 ± 0.109	0.661 ± 0.125	0.719 ± 0.136
2.5D-ResUNet	0.537 ± 0.112	0.692 ± 0.095	0.688 ± 0.127^†^	0.720 ± 0.116
2.5D-AttUNet	0.544 ± 0.124	0.696 ± 0.109	0.715 ± 0.120	0.703 ± 0.139
3D-UNet	0.569 ± 0.107	0.719 ± 0.092	0.664 ± 0.131	0.812 ± 0.095
3D-Swin UNETR	0.548 ± 0.104	0.702 ± 0.088	0.645 ± 0.122	0.795 ± 0.092

^*^Significant difference in PR between 3D-UNet and 2D-ResUNet (*p*-value < 0.05)

^†^Significant difference in PR between 3D-UNet and 2.5D-ResUNet (*p*-value < 0.05)

**Fig. 3:**
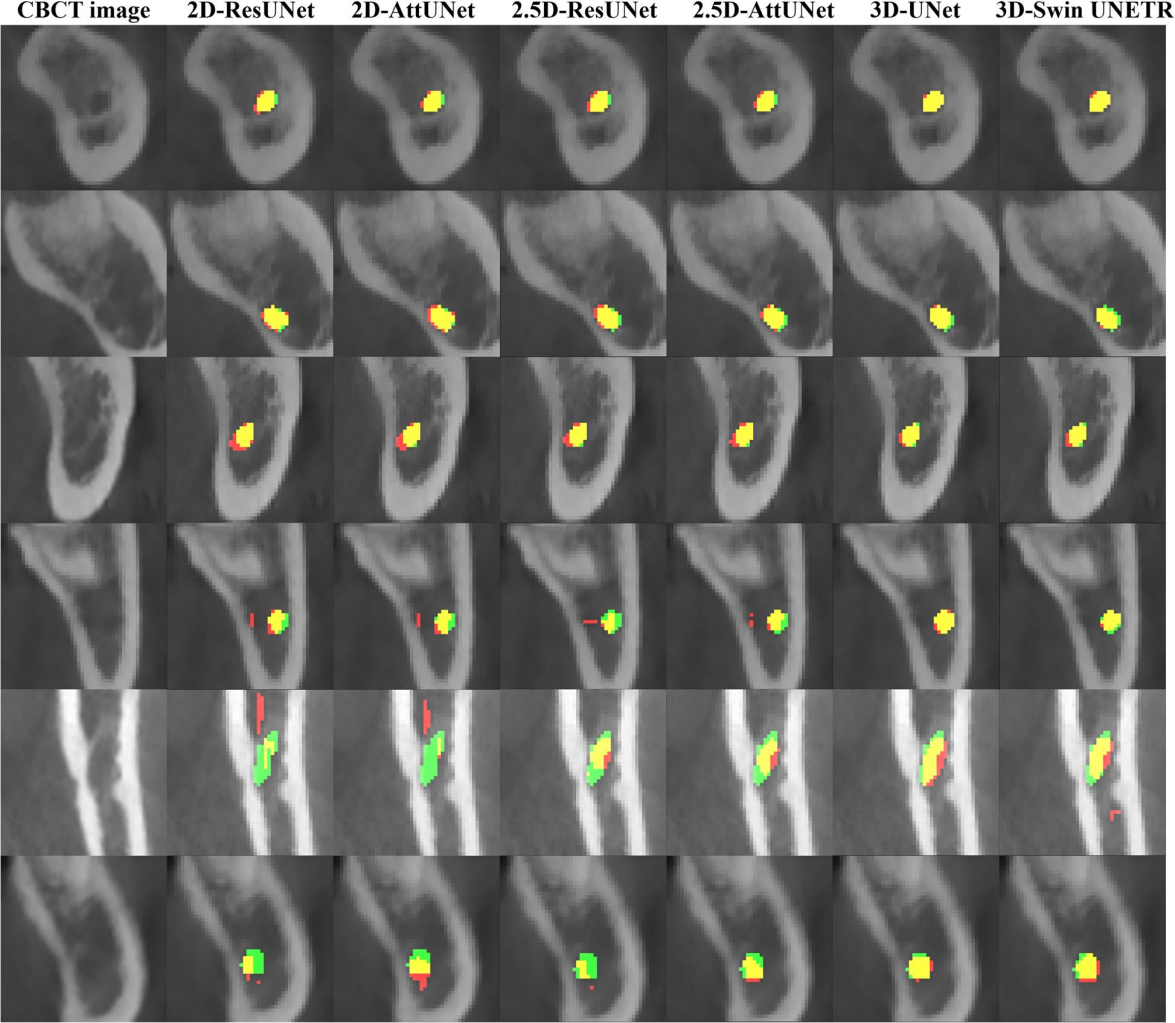
Representative segmentation of mandibular canals from the 2D, 2.5D, and 3D segmentation networks on the public test dataset. The yellow, green, and red areas represent true positives, false negatives, and false positives, respectively

**Fig. 4:**
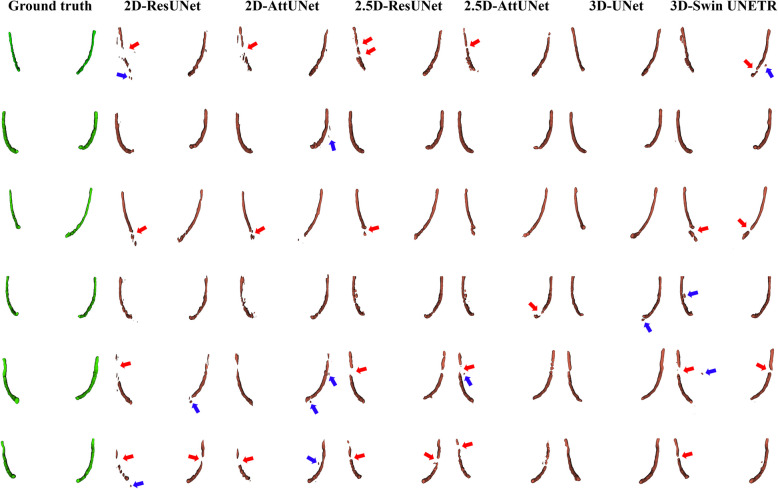
3D reconstruction results of the mandibular canals from the 2D, 2.5D, and 3D segmentation networks on the public test dataset. The red and blue arrows indicate disconnections and false segmentations, respectively

**Fig. 5:**
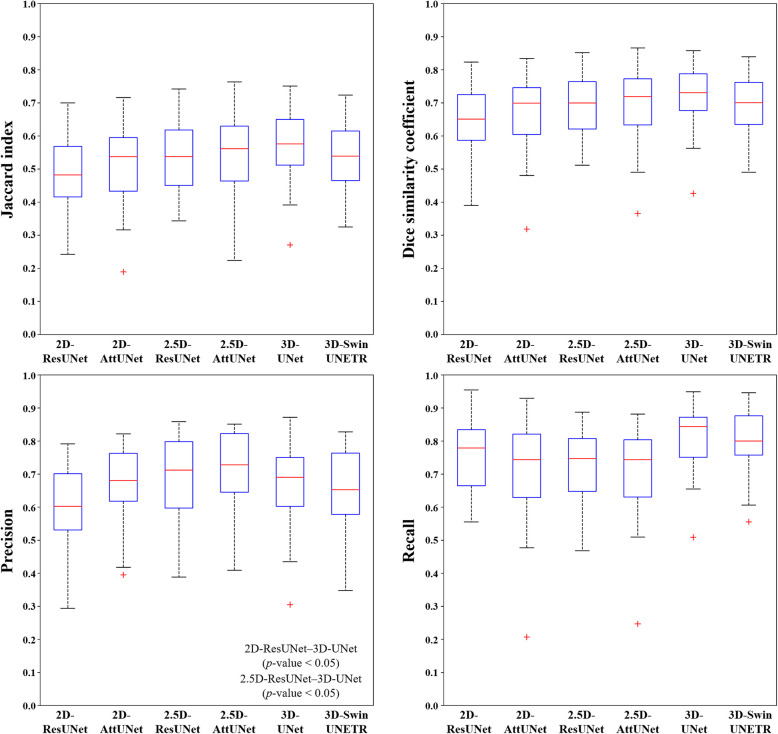
Boxplots of the Jaccard index, Dice similarity coefficient, precision, and recall values from 2D-ResUNet, 2D-AttUNet, 2.5D-ResUNet, 2.5D-AttUNet, 3D-UNet, and 3D-Swin UNETR on the public test dataset. A significant difference in PR between 3D-UNet and 2D-ResUNet (*p*-value < 0.05). A significant difference in PR between 3D-UNet and 2.5D-ResUNet (*p*-value < 0.05)

Figure 3 shows representative results from the 2D, 2.5D, and 3D segmentation networks. 3D-UNet exhibited the most accurate segmentation results for the MC in low-contrast CBCT images, with more true positives (yellow), fewer false positives (red), and fewer false negatives (green) than the 2D and 2.5D segmentation networks. The 2D segmentation networks showed more false positives and negatives for MCs with low contrast than the 2.5D segmentation networks. In the 3D reconstruction results, the 3D segmentation networks demonstrated better predictions with fewer false positives and false negatives on the entire MC (Fig. 4) and improved structural connectivity and boundary details for the MC from the mental foramen to the mandibular foramen compared to the 2D and 2.5D segmentation networks (Fig. 7a). 3D-UNet achieved higher segmentation performances than the other networks, with a smaller dispersion of data and shorter whiskers (Fig. 5).

**Fig. 6:**
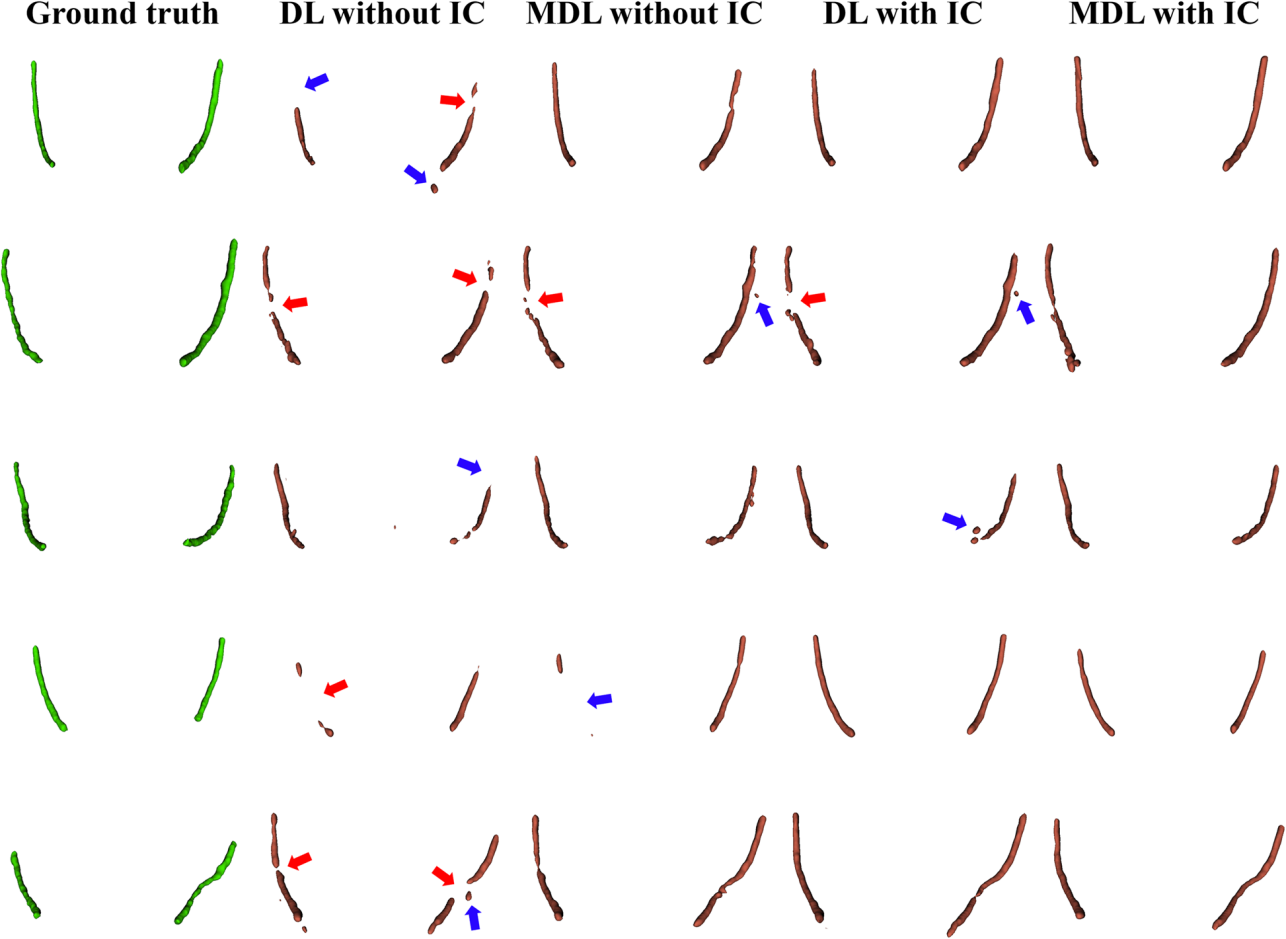
3D reconstruction results of the mandibular canals from 3D-UNet using the image cropping technique and segmentation loss functions on the public test dataset. The red and blue arrows indicate disconnections and false segmentations, respectively. DL, IC, and MDL represent the Dice loss, image cropping technique, and multi-planar Dice loss, respectively

**Fig. 7:**
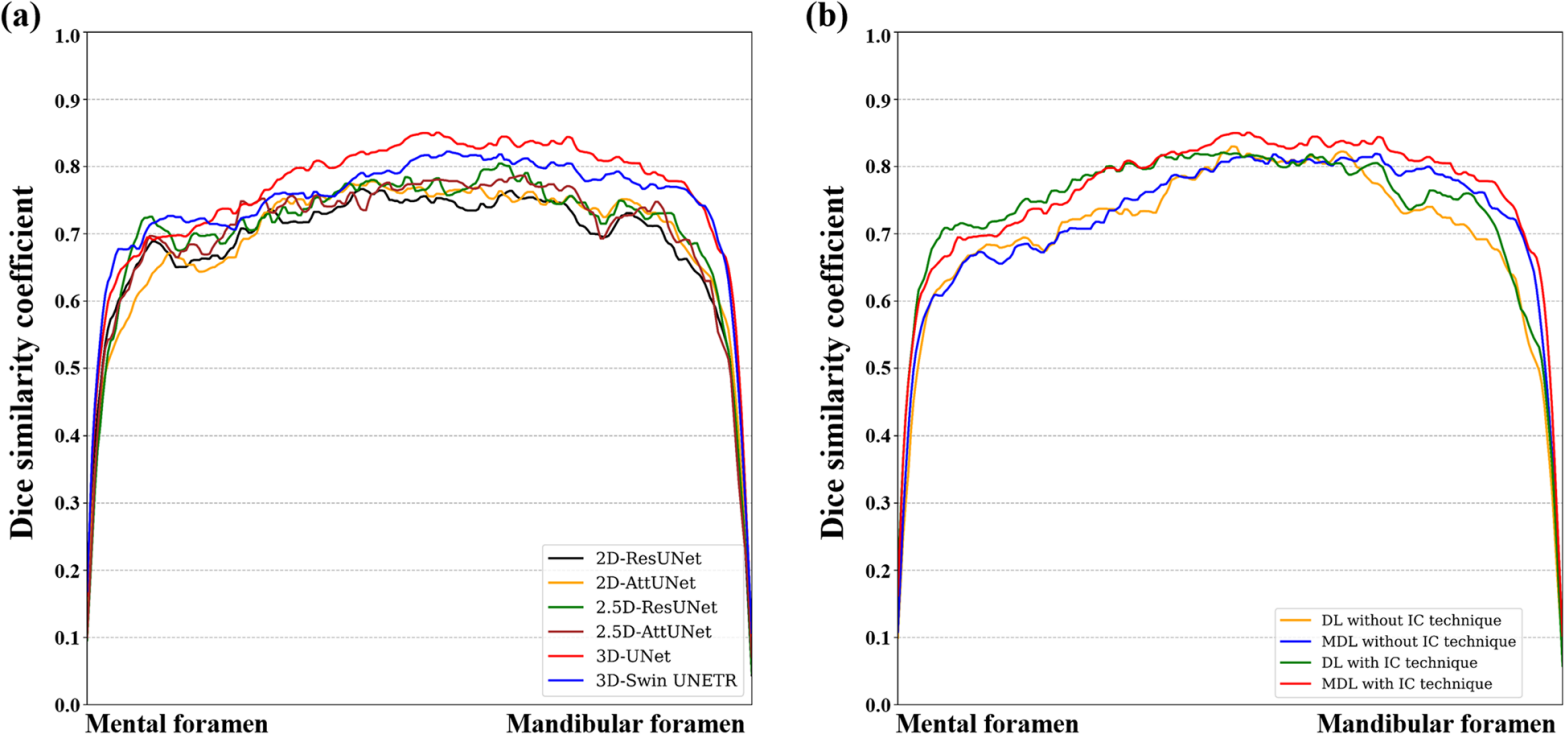
Line plots of the Dice similarity coefficient (DSC) from the mental foramen to the mandibular foramen on the public test dataset. **a** Line plots of the DSC from 2D-ResUNet, 2D-AttUNet, 2.5D-ResUNet, 2.5D-AttUNet, 3D-UNet, and 3D-Swin UNETR. **b** Line plots of the DSC from 3D-UNet in the ablation studies

We performed ablation studies to compare the IC technique and segmentation loss functions. All segmentation networks showed improvements in segmentation performance with the IC technique, especially in reducing false negatives and improving the continuity of the MC (Table 3). In the 3D segmentation networks, $MDL$ outperformed $DL$ in terms of JC, DSC, and RC (Table 4). Also, 3D-UNet with the IC technique and $MDL$ demonstrated improved boundary details (Fig. 6) and structural connectivity (Fig. 7b) of the MC from the mental foramen to the mandibular foramen.

**Table 3:** Segmentation performances in terms of Jaccard index (JI), Dice similarity coefficient (DSC), precision (PR), and recall (RC) for the mandibular canals from 2D, 2.5D, and 3D segmentation networks using the image cropping (IC) technique on the public test dataset. The results of 2D-ResUNet and 2D-AttUNet are obtained by training coronal CBCT images

Methods	JI	DSC	PR	RC
Without the IC technique				
2D-ResUNet	0.471 ± 0.114	0.632 ± 0.102	0.614 ± 0.125	0.678 ± 0.136
2D-AttUNet	0.494 ± 0.123	0.652 ± 0.108	0.643 ± 0.135	0.684 ± 0.127
2.5D-ResUNet	0.535 ± 0.118	0.690 ± 0.099	0.722 ± 0.132	0.687 ± 0.131
2.5D-AttUNet	0.535 ± 0.131	0.688 ± 0.112	0.722 ± 0.136	0.685 ± 0.145
3D-UNet	0.554 ± 0.132	0.703 ± 0.116	0.707 ± 0.135	0.738 ± 0.112
3D-Swin UNETR	0.510 ± 0.113	0.668 ± 0.099	0.633 ± 0.138	0.736 ± 0.160
With the IC technique				
2D-ResUNet	0.476 ± 0.125	0.635 ± 0.117	0.592 ± 0.132	0.702 ± 0.127
2D-AttUNet	0.516 ± 0.117	0.673 ± 0.103	0.629 ± 0.123	0.737 ± 0.102
2.5D-ResUNet	0.537 ± 0.112	0.692 ± 0.095	0.688 ± 0.127	0.720 ± 0.116
2.5D-AttUNet	0.544 ± 0.124	0.696 ± 0.109	0.715 ± 0.120	0.703 ± 0.139
3D-UNet	0.569 ± 0.107	0.719 ± 0.092	0.664 ± 0.131	0.812 ± 0.095
3D-Swin UNETR	0.548 ± 0.104	0.702 ± 0.088	0.645 ± 0.122	0.795 ± 0.092

**Table 4:** Segmentation performances in terms of Jaccard index (JI), Dice similarity coefficient (DSC), precision (PR), and recall (RC) for the mandibular canals from 3D-UNet and 3D-Swin UNETR using the image cropping (IC) technique and segmentation loss functions (Dice and multi-planar Dice losses; DL and MDL, respectively) on the public test dataset

Methods	JI	DSC	PR	RC
DL without the IC technique				
3D-UNet	0.545 ± 0.136	0.699 ± 0.121	0.730 ± 0.136	0.719 ± 0.177
3D-Swin UNETR	0.508 ± 0.118	0.666 ± 0.103	0.676 ± 0.136	0.682 ± 0.128
DL with the IC technique				
3D-UNet	0.567 ± 0.108	0.717 ± 0.091	0.716 ± 0.131	0.751 ± 0.127
3D-Swin UNETR	0.526 ± 0.120	0.682 ± 0.099	0.649 ± 0.127	0.743 ± 0.117
MDL without the IC technique				
3D-UNet	0.554 ± 0.132	0.703 ± 0.116	0.707 ± 0.135	0.738 ± 0.112
3D-Swin UNETR	0.510 ± 0.113	0.668 ± 0.099	0.633 ± 0.138	0.736 ± 0.160
MDL with the IC technique				
3D-UNet	0.569 ± 0.107	0.719 ± 0.092	0.664 ± 0.131	0.812 ± 0.095
3D-Swin UNETR	0.548 ± 0.104	0.702 ± 0.088	0.645 ± 0.122	0.795 ± 0.092

**Fig. 8:**
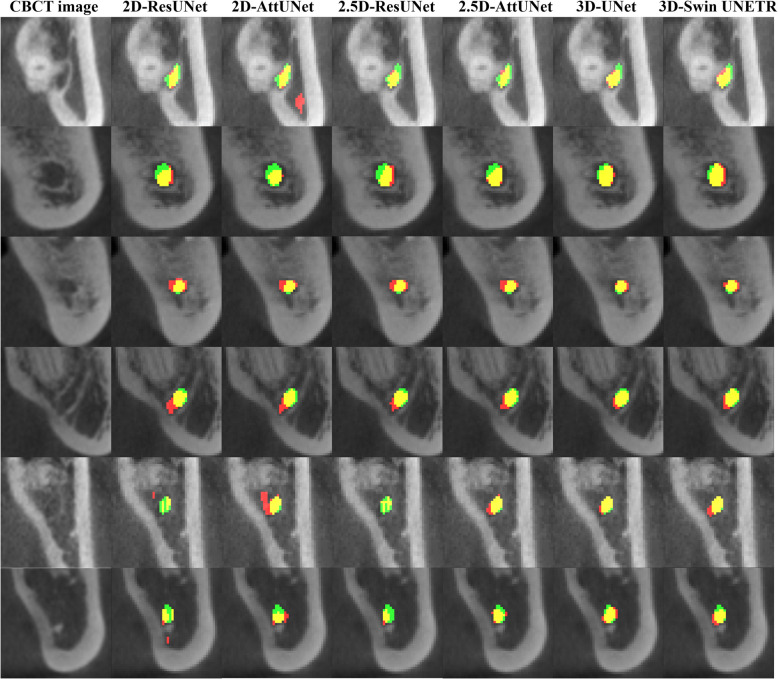
Representative segmentation results for the mandibular canals from the 2D, 2.5D, and 3D segmentation networks on the external test dataset. The yellow, green, and red areas represent true positives, false negatives, and false positives, respectively

**Fig. 9:**
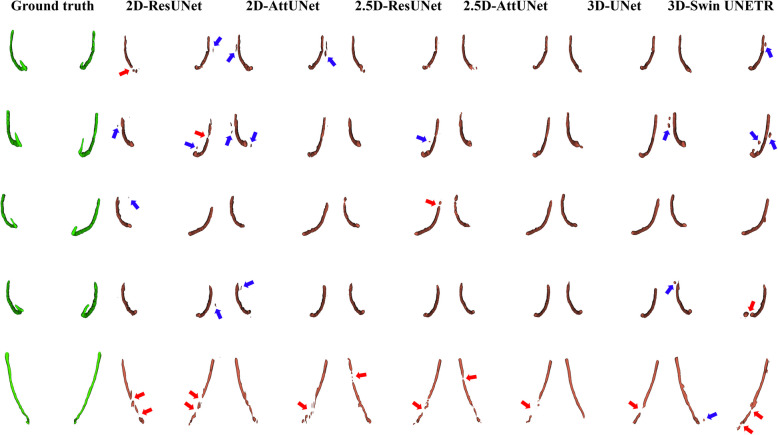
3D reconstruction results of the mandibular canals from the 2D, 2.5D, and 3D segmentation networks on the external test dataset. The red and blue arrows indicate disconnections and false segmentations, respectively

**Fig. 10:**
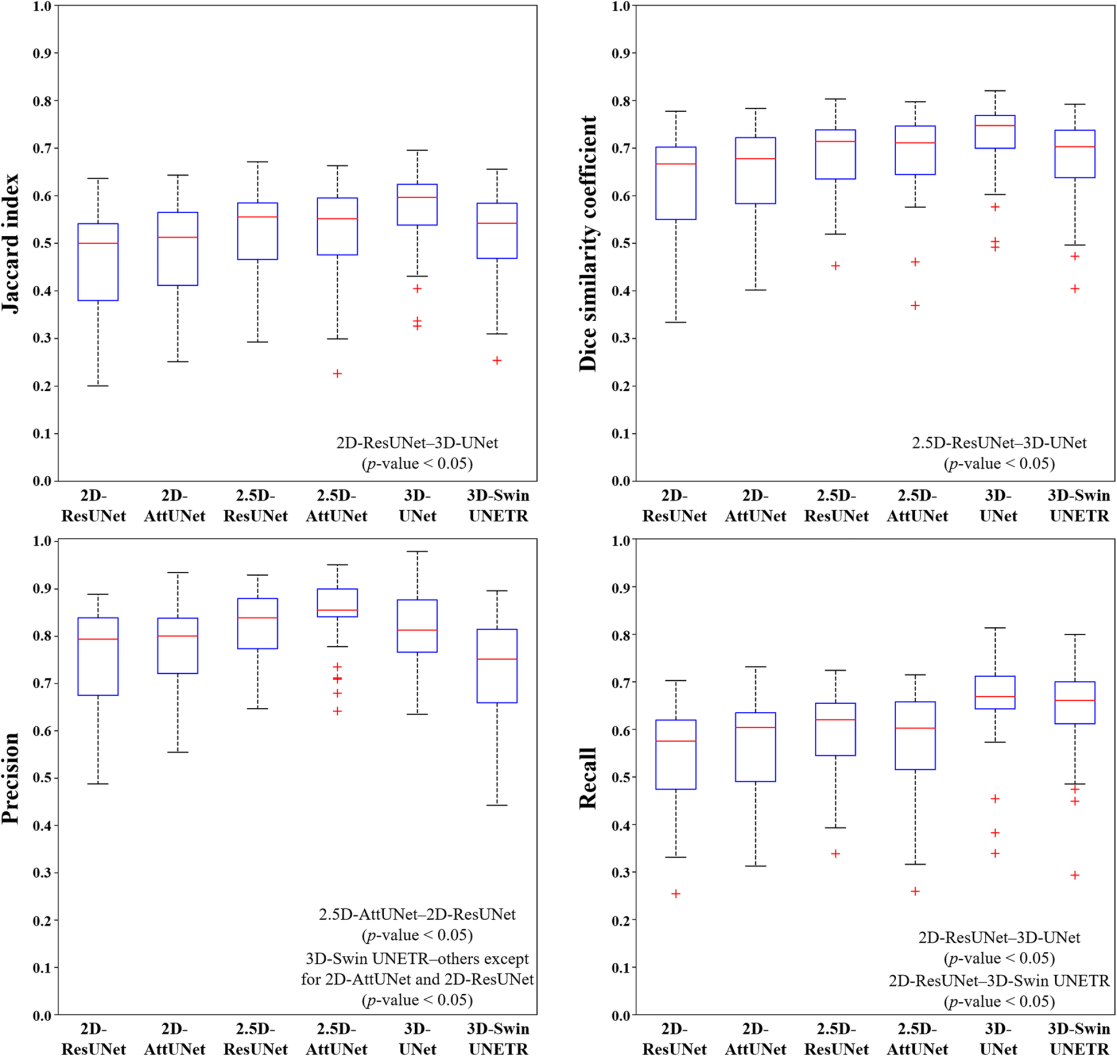
Boxplots of the Jaccard index, Dice similarity coefficient, precision, and recall values from 2D-ResUNet, 2D-AttUNet, 2.5D-ResUNet, 2.5D-AttUNet, 3D-UNet, and 3D-Swin UNETR on the external test dataset. A significant difference in JI between 3D-UNet and 2D-ResUNet (*p*-value < 0.05). A significant difference in DSC between 3D-UNet and 2.5D-ResUNet (*p*-value < 0.05). A significant difference in PR between 2.5D-AttUNet and 2D-ResUNet (*p*-value < 0.05). A significant difference in PR between 3D-Swin UNETR and others except for 2D-AttUNet and 2D-ResUNet (*p*-value < 0.05). A significant difference in RC between 3D-UNet and 2D-ResUNet (*p*-value < 0.05). A significant difference in RC between 3D-Swin UNETR and 2D-ResUNet (*p*-value < 0.05)

**Fig. 11:**
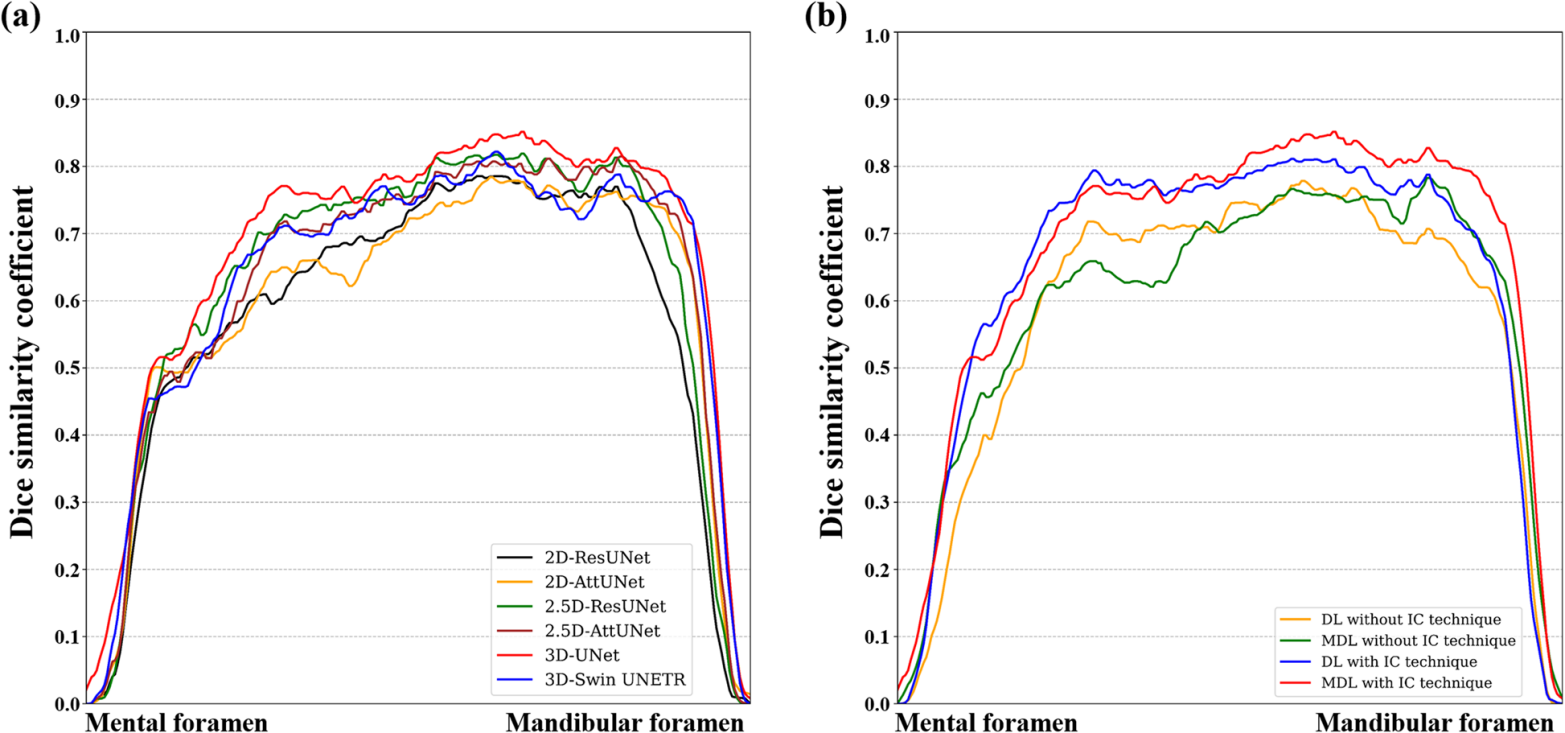
Line plots of the Dice similarity coefficient (DSC) from the mental foramen to the mandibular foramen on the external test dataset. **a** Line plots of the DSC from 2D-ResUNet, 2D-AttUNet, 2.5D-ResUNet, 2.5D-AttUNet, 3D-UNet, and 3D-Swin UNETR. **b** Line plots of the DSC from 3D-UNet in the ablation studies

On the external test dataset, 3D-UNet showed the best segmentation performance, outperforming the 2D and 2.5D segmentation networks (Table 5). 3D-UNet achieved 0.564 ± 0.092, 0.716 ± 0.081, and 0.652 ± 0.103 in terms of JC, DSC, and RC, respectively. The 2.5D-AttUNet showed the best PR value of 0.843 ± 0.077 and outperformed the 2D segmentation networks in terms of JC, DSC, and PR (Table 5). Figure 8 shows representative results for the MC from 2D, 2.5D, and 3D segmentation networks on the external test dataset. 3D-UNet exhibited the most accurate segmentation results for MCs from low-contrast CBCT images, with more true positives (yellow), fewer false positives (red), and fewer false negatives (green) than the 2D and 2.5D segmentation networks. The 2D segmentation networks showed more false positives and negatives for the MCs in low-contrast CBCT images than the 2.5D segmentation networks. In the 3D reconstruction results, the 3D segmentation networks demonstrated better predictions with fewer false positives and negatives on the entire MC (Fig. 9) and improved structural connectivity and boundary details for the MC from the mental foramen to the mandibular foramen, compared with the 2D and 2.5D segmentation networks (Fig. 11a). 3D-UNet achieved higher segmentation performances on the external test dataset than the other networks, with a smaller dispersion of data and shorter whiskers (Fig. 10). In the external test dataset, 3D-UNet with the IC technique and $MDL$ demonstrated improved boundary details and structural connectivity of the MC from the mental foramen to the mandibular foramen (Fig. 11b). The comparison of the segmentation performance on the external test dataset by the different ensemble methods and 2D segmentation networks trained on the axial, sagittal, and coronal planes are shown in Supplementary Tables S3 and S4, respectively. Ablation studies for the IC technique and segmentation loss functions on the external test dataset are provided in Supplementary Tables S5 and S6.

**Table 5:** Segmentation performances in terms of the Jaccard index (JI), Dice similarity coefficient (DSC), precision (PR), and recall (RC) for mandibular canals from 2D, 2.5D, and 3D segmentation networks on the external test dataset

Segmentation networks	JI	DSC	PR	RC
2D-ResUNet	0.467 ± 0.107^*^	0.629 ± 0.106^†^	0.758 ± 0.099^‡^	0.542 ± 0.111^††,^^‡‡^
2D-AttUNet	0.488 ± 0.104	0.649 ± 0.100	0.775 ± 0.096	0.565 ± 0.112
2.5D-ResUNet	0.526 ± 0.093	0.684 ± 0.085	0.820 ± 0.079	0.591 ± 0.094
2.5D-AttUNet	0.526 ± 0.100	0.683 ± 0.094	0.843 ± 0.077	0.582 ± 0.107
3D-UNet	0.564 ± 0.092	0.716 ± 0.081	0.812 ± 0.087	0.652 ± 0.103
3D-Swin UNETR	0.515 ± 0.104	0.673 ± 0.098	0.722 ± 0.117^**^	0.641 ± 0.103

^*^Significant difference in JI between 3D-UNet and 2D-ResUNet (*p*-value < 0.05)

^†^Significant difference in DSC between 3D-UNet and 2.5D-ResUNet (*p*-value < 0.05)

^‡^Significant difference in PR between 2.5D-AttUNet and 2D-ResUNet (*p*-value < 0.05)

^*^^*^Significant difference in PR between 3D-Swin UNETR and others except for 2D-AttUNet and 2D-ResUNet (*p*-value < 0.05)

^††^Significant difference in RC between 3D-UNet and 2D-ResUNet (*p*-value < 0.05)

^‡‡^Significant difference in RC between 3D-Swin UNETR and 2D-ResUNet (*p*-value < 0.05)

## Discussion

Accurate identification of the MC in CBCT images is essential in planning maxillofacial surgeries, such as dental implant placement and third molar extractions, to prevent inadvertent injury to the inferior alveolar nerve [4–6]. However, in cases of anatomical variations of the MC, it is challenging for even dental clinicians to identify the MC in low-contrast CBCT images [11, 12]. Also, the manual segmentation process of the MC in CBCT images is laborious and time-consuming. Therefore, automatic segmentation methods are needed to reduce the workload of dental clinicians.

In previous studies, various deep-learning models were designed to automatically segment the MC in CBCT images [11, 17–19]. Jeoun et al. [11] achieved 0.80 ± 0.06, 0.87 ± 0.05, 0.89 ± 0.06, and 0.88 ± 0.06 in terms of JI, DSC, PR, and RC for the MC segmentation in CBCT volumes using a 3D segmentation network. They also reported JI, DSC, PR, and RC values of 0.77 ± 0.07, 0.84 ± 0.07, 0.85 ± 0.07, and 0.84 ± 0.08, respectively, using a 2D segmentation network. Jinxuan et al. [17] proposed a ViT-based 3D network for MC segmentation in a CBCT volume. They achieved JI and DSC values of 0.73 and 0.84, respectively, for MC segmentation. Lahoud et al. [18] developed a patch-wise 3D U-Net that achieved JI, DSC, PR, and RC values of 0.64 ± 0.08, 0.79 ± 0.11, 0.78 ± 0.12, and 0.79 ± 0.11, respectively, for MC segmentation in CBCT volumes. DU et al. [19] proposed a 3D attention-based U-Net for MC segmentation in a CBCT volume and reported JI, DSC, PR, and RC values of 0.75, 0.86, 0.84, and 0.89, respectively. Those 2D and 3D segmentation networks efficiently and accurately segmented the MC in CBCT images, and they could thus be alternatives to manual segmentation. However, although previous studies proposed methods for segmenting the MC in CBCT images using 2D or 3D segmentation networks based on deep learning, it was still unclear which kind of segmentation network (2D, 2.5D, or 3D segmentation) performed best on various CBCT datasets. Therefore, we compared the MC segmentation performances of 2D, 2.5D, and 3D segmentation networks using public and external datasets. As far as we know, no previous studies have been reported to compare the segmentation performance of the MC among 2D, 2.5D, and 3D segmentation networks on public and external datasets.

We compared the performance of 2D, 2.5D, and 3D segmentation networks for the MC in CBCT images. The 3D-UNet exhibited superior performance in terms of JI, DSC, and RC compared to 2D and 2.5D segmentation networks on both public and external test datasets. This superior performance can be attributed to the ability of 3D segmentation networks to learn spatially continuous features across the entire CBCT volumes, preserving structural continuity and reducing false segmentations. 2.5D-AttUNet showed the best PR value and outperformed the 2D segmentation networks in terms of JC, DSC, and PR. The 2.5D segmentation networks, trained using only 2D CBCT images, utilized 3D volumetric information by combining segmentation results from the axial, sagittal, and coronal planes using an ensemble method [20, 21]. This approach resulted in more accurate segmentation than traditional 2D segmentation networks while still needing only the memory capacity of a 2D segmentation network. Although the 2.5D segmentation networks achieved higher segmentation performance than the 2D segmentation networks, there were false positives and negatives in MC areas with ambiguous or unclear cortical bone layers due to the lack of 3D volumetric context information. Furthermore, the 2D and 2.5D segmentation networks frequently showed disconnections in the MC segmentation results, which may pose challenges for clinical applications such as dental implant placement and third molar extractions.

By learning the 3D volumetric context information for the entire MC, the 3D segmentation networks showed superior segmentation performance with improved structural continuity, smooth boundaries, and more consistent accuracy from the mental to the mandibular foramen areas in CBCT volumes. The 3D segmentation networks demonstrate higher practical applicability in clinical workflows compared to the 2D and 2.5D segmentation networks, minimizing the need for manual annotation and enhancing the reliability of automated canal segmentation in CBCT volumes. However, their higher computational and memory requirements remain a consideration for real-time or resource-constrained environments. These findings underscore the importance of selecting the appropriate segmentation approach based on the specific application requirements, balancing accuracy, continuity, and computational resources [20, 21].

We observed that 3D-Swin UNETR, which is based on the ViT approach, was not always better than 3D-UNet in segmenting the MC. Two factors could contribute to the superior segmentation performance of 3D-UNet over 3D-Swin UNETR: (1) In our task, MC segmentation in CBCT images, local patterns of the MC are more important than global long-range information relationships between anatomical structures. The diameter of MC is relatively small compared with other anatomical structures within the mandible. In a previous manual analysis, the mean diameter and length of the MC in CBCT images were measured to be 2.59 mm and 65.71 mm on the right and 2.71 mm and 60.03 mm on the left [30]. CNNs use convolutional operations with local receptive fields, which allow the network to focus on small, localized regions of the input [31, 32]. This architecture design helps a deep-learning model effectively learn and capture detailed local patterns such as edges, textures, and small shapes [31]. (2) The ViT approach, lacking inductive biases such as locality and translation invariance of CNNs, sometimes requires substantially larger training datasets and more task-specific tuning of hyperparameters to learn local patterns and features and achieve optimal segmentation performance [15, 32, 33]. Although we used 122 CBCT volumes for network training, the size of the training dataset is not guaranteed to be sufficient to optimize the 3D-Swin UNETR.

We performed ablation studies to compare the IC technique and segmentation loss functions. All segmentation networks showed improvements in segmentation performance with the IC technique, especially in reducing false negatives and improving the continuity of the MC. The IC technique has two advantages: (1) the IC technique increased the training dataset by allowing the left and right sides to be used as a separate training dataset. Therefore, we effectively doubled the available training data without requiring additional labeled datasets, enhancing the segmentation performance of all segmentation networks; (2) the IC technique ensures uniformity in spatial orientation, which enables segmentation networks to learn more robust and uniform feature representation by eliminating inconsistencies in spatial representation. In the 3D segmentation networks, $MDL$ outperformed $DL$ in terms of JC, DSC, and RC, with improvements in boundary details and structural connectivity of the MC. $MDL$ provided global structural continuity from three anatomical projection maps of the MC volume, which reduces disconnections in the MC segmentation and enhances the overall connectivity of the MC by enforcing consistency across the axial, sagittal, and coronal planes [11]. By integrating the IC technique and $MDL$ into the segmentation networks, we achieved more consistent and accurate MC segmentation in CBCT volumes. This advancement is beneficial for clinical applications where high segmentation fidelity of the MC is essential for dental implant placement and third molar extractions in CBCT images.

We compared the performance of 2D, 2.5D, and 3D segmentation networks for the MC in CBCT images. The 3D-UNet exhibited superior performance in terms of JI, DSC, and RC compared to 2D and 2.5D segmentation networks on both public and external test datasets. Traditional loss functions such as binary cross-entropy or single-plane Dice loss often struggle to maintain segmentation consistency across different planes, leading to discontinuities or misalignment in 3D structures. To overcome this, we introduced a multi-planar Dice similarity coefficient (DSC) loss, which computes the segmentation loss across multiple views of axial, sagittal, and coronal.

The generalization problem is a fundamental challenge in deploying deep-learning models in real-world applications, and deep-learning models must ensure consistent segmentation performance across different datasets acquired from multiple centers or devices [34–36]. This consistency is essential for maintaining confidence and robustness in deep-learning model predictions and making informed decisions based on the outputs. In this study, we evaluated the segmentation performance of 2D, 2.5D, and 3D segmentation networks on an external test dataset to validate their generalizability and robustness. All the segmentation networks showed consistent segmentation performance on the external test dataset, and 3D-UNet achieved the best MC segmentation performance among them. The major finding in this study is that 3D segmentation networks demonstrated more accurate and robust segmentation performance for MCs than 2D and 2.5D segmentation networks on both public and external datasets under the same GPU memory capacity.

This study has several limitations. First, we evaluated the performance of 2D, 2.5D, and 3D segmentation networks on 31 public and 30 external test datasets. To further improve the segmentation performance, the segmentation networks need to be trained and evaluated using larger datasets from more patients receiving various dental treatments using CBCT devices and scanning parameters in multiple centers. Second, further research is needed to carefully perform task-specific tuning of the hyperparameters in ViT models to obtain optimal MC segmentation performance in CBCT images [37]. In future works, we will compare the segmentation performance according to age and sex, different CBCT image quality, and various complex cases with diseases. Additionally, we plan to apply improved 3D data augmentation [38] and diffusion-based image augmentation methods to compensate for the lack of CBCT datasets when training ViT models [39, 40].

## Conclusions

In this study, we compared the segmentation performance for the MC in CBCT images using 2D, 2.5D, and 3D segmentation networks under the same GPU memory capacity. The 3D-UNet demonstrated superior segmentation performance for the MC by learning 3D volumetric context information for the entire MC in CBCT volumes. Also, the IC technique and *MDL* further improved the boundary details and structural connectivity of the MC from the mental foramen to the mandibular foramen. Therefore, 3D-UNet could contribute to accurate and robust segmentation of the MC in CBCT volumes and minimize the need for manual annotation for the pre-operative planning of dental implant surgeries and third molar extractions.

## Supplementary Information

Supplementary Material 1.

## Data Availability

This study was performed with approval from the Institutional Review Board (IRB) of Seoul National University Dental Hospital (ERI18001). The IRB of Seoul National University Dental Hospital approved the waiver for informed consent because this was a retrospective study. The study was performed in accordance with the Declaration of Helsinki. The data used in this study are not publicly available due to patient privacy and therefore cannot be shared.
