# Supplementary material for: Comparison of 2D, 2.5D, and 3D segmentation networks for mandibular canals in CBCT images: a study on public and external datasets
Source: BMC Oral Health. 2025 Jul 8;25:1126. doi: 10.1186/s12903-025-06483-4 (PMC12235980; doi:10.1186/s12903-025-06483-4)
Supplement: Supplementary file 1 — Supplementary Material 1. [file 12903_2025_6483_MOESM1_ESM.docx]

Supplementary Materials

| Ensemble  methods | JI | DSC | PR | RC |
| --- | --- | --- | --- | --- |
| **Unanimous** |  |  |  |  |
| 2.5D-ResUNet | 0.411±0.156 | 0.565±0.158 | 0.765±0.151 | 0.474±0.174 |
| 2.5D-AttUNet | 0.419±0.154 | 0.573±0.162 | **0.791±0.151** | 0.473±0.169 |
| **Affirmative** |  |  |  |  |
| 2.5D-ResUNet | 0.467±0.101 | 0.631±0.093 | 0.503±0.109 | **0.872±0.062** |
| 2.5D-AttUNet | 0.504±0.101 | 0.665±0.090 | 0.550±0.111 | 0.863±0.063 |
| **Majority** |  |  |  |  |
| 2.5D-ResUNet | 0.537±0.112 | 0.692±0.095 | 0.688±0.127 | 0.720±0.116 |
| 2.5D-AttUNet | **0.544±0.124** | **0.696±0.109** | 0.715±0.120 | 0.703±0.139 |

**Table S1.** Segmentation performances in terms of Jaccard coefficient (JI), Dice similarity coefficient (DSC), precision (PR), and recall (RC) for the mandibular canals from 2.5D segmentation networks using different ensemble methods (unanimous, affirmative, and majority) on the public test dataset.

**Table S2.** Segmentation performances in terms of Jaccard coefficient (JI), Dice similarity coefficient (DSC), precision (PR), and recall (RC) for the mandibular canals from 2D segmentation networks using different planes on the public test dataset.

| Methods | JI | DSC | PR | RC |
| --- | --- | --- | --- | --- |
| **Axial plane** |  |  |  |  |
| 2D-ResUNet | 0.431±0.129 | 0.591±0.132 | 0.548±0.124 | 0.656±0.164 |
| 2D-AttUNet | 0.441±0.135 | 0.599±0.137 | 0.631±0.138 | 0.595±0.161 |
| **Coronal plane** |  |  |  |  |
| 2D-ResUNet | 0.476±0.125 | 0.635±0.117 | 0.592±0.132 | 0.702±0.127 |
| 2D-AttUNet | 0.516±0.117 | 0.673±0.103 | 0.629±0.123 | **0.737±0.102** |
| **Sagittal plane** |  |  |  |  |
| 2D-ResUNet | 0.518±0.109 | 0.675±0.097 | **0.676±0.122** | 0.699±0.127 |
| 2D-AttUNet | **0.521±0.123** | **0.676±0.109** | 0.661±0.125 | 0.719±0.136 |

| Ensemble  methods | JI | DSC | PR | RC |
| --- | --- | --- | --- | --- |
| **Unanimous** |  |  |  |  |
| 2.5D-ResUNet | 0.402±0.113 | 0.564±0.120 | 0.900±0.054 | 0.420±0.117 |
| 2.5D-AttUNet | 0.392±0.124 | 0.551±0.137 | **0.911±0.054** | 0.408±0.130 |
| **Affirmative** |  |  |  |  |
| 2.5D-ResUNet | 0.479±0.101 | 0.642±0.095 | 0.577±0.120 | **0.734±0.068** |
| 2.5D-AttUNet | 0.511±0.094 | 0.671±0.088 | 0.624±0.112 | 0.734±0.074 |
| **Majority** |  |  |  |  |
| 2.5D-ResUNet | **0.526±0.093** | **0.684±0.085** | 0.820±0.079 | 0.591±0.094 |
| 2.5D-AttUNet | 0.526±0.100 | 0.683±0.094 | 0.843±0.077 | 0.582±0.107 |

**Table S3.** Segmentation performances in terms of Jaccard coefficient (JI), Dice similarity coefficient (DSC), precision (PR), and recall (RC) for the mandibular canals from 2.5D segmentation networks using different ensemble methods (unanimous, affirmative, and majority) on the external test dataset.

**Table S4.** Segmentation performances in terms of Jaccard coefficient (JI), Dice similarity coefficient (DSC), precision (PR), and recall (RC) for the mandibular canals from 2D segmentation networks using different planes on the external test dataset.

| Models | JI | DSC | PR | RC |
| --- | --- | --- | --- | --- |
| **Axial plane** |  |  |  |  |
| 2D-ResUNet | 0.449±0.089 | 0.614±0.084 | 0.653±0.111 | 0.590±0.092 |
| 2D-AttUNet | 0.442±0.095 | 0.606±0.097 | 0.710±0.105 | 0.535±0.100 |
| **Coronal plane** |  |  |  |  |
| 2D-ResUNet | 0.501±0.112 | 0.660±0.107 | 0.720±0.139 | 0.613±0.090 |
| 2D-AttUNet | **0.516±0.112** | **0.673±0.106** | 0.737±0.124 | **0.625±0.106** |
| **Sagittal plane** |  |  |  |  |
| 2D-ResUNet | 0.467±0.107 | 0.629±0.106 | 0.758±0.099 | 0.542±0.111 |
| 2D-AttUNet | 0.488±0.104 | 0.649±0.100 | **0.775±0.096** | 0.565±0.112 |

| Models | JI | DSC | PR | RC |
| --- | --- | --- | --- | --- |
| **Without the IC technique** |  |  |  |  |
| 2D-ResUNet | 0.495±0.111 | 0.654±0.104 | 0.740±0.119 | 0.590±0.103 |
| 2D-AttUNet | 0.496±0.118 | 0.654±0.114 | 0.723±0.128 | **0.601±0.114** |
| 2.5D-ResUNet | **0.519±0.098** | **0.678±0.091** | 0.818±0.088 | 0.583±0.097 |
| 2.5D-AttUNet | 0.518±0.106 | 0.675±0.102 | **0.841±0.076** | 0.573±0.115 |
| 3D-UNet | 0.482±0.124 | 0.639±0.127 | 0.775±0.130 | 0.561±0.137 |
| 3D-Swin UNETR | 0.451±0.098 | 0.615±0.100 | 0.718±0.087 | 0.549±0.120 |
| **With IC the technique** |  |  |  |  |
| 2D-ResUNet | 0.501±0.112 | 0.660±0.107 | 0.720±0.139 | 0.613±0.090 |
| 2D-AttUNet | 0.516±0.112 | 0.673±0.106 | 0.737±0.124 | 0.625±0.106 |
| 2.5D-ResUNet | 0.526±0.093 | 0.684±0.085 | 0.820±0.079 | 0.591±0.094 |
| 2.5D-AttUNet | 0.526±0.100 | 0.683±0.094 | **0.843±0.077** | 0.582±0.107 |
| 3D-UNet | **0.564±0.092** | **0.716±0.081** | 0.812±0.087 | **0.652±0.103** |
| 3D-Swin UNETR | 0.515±0.104 | 0.673±0.098 | 0.722±0.117 | 0.641±0.103 |

**Table S5.** Segmentation performances in terms of Jaccard coefficient (JI), Dice similarity coefficient (DSC), precision (PR), and recall (RC) for the mandibular canals from 2D, 2.5D, and 3D segmentation networks using the image cropping (IC) technique on the external test dataset. The results of 2D-ResUNet and 2D-AttUNet are obtained by training coronal CBCT images.

**Table S6.** Segmentation performances in terms of Jaccard coefficient (JI), Dice similarity coefficient (DSC), precision (PR), and recall (RC) for the mandibular canals from 3D-UNet and 3D-Swin UNETR using the image cropping (IC) technique and segmentation loss functions (Dice and multi-planar Dice losses) on the external test dataset.

| Methods | JI | DSC | PR | RC |
| --- | --- | --- | --- | --- |
| **DL without the IC**  **technique** |  |  |  |  |
| 3D-UNet | 0.473±0.111 | 0.634±0.108 | 0.784±0.078 | 0.545±0.130 |
| 3D-Swin UNETR | 0.455±0.097 | 0.619±0.096 | 0.719±0.102 | 0.553±0.115 |
| **DL with the IC technique** |  |  |  |  |
| 3D-UNet | 0.531±0.085 | 0.689±0.083 | **0.827±0.073** | 0.601±0.098 |
| 3D-Swin UNETR | 0.492±0.108 | 0.652±0.102 | 0.698±0.130 | 0.619±0.096 |
| **MDL without the IC technique** |  |  |  |  |
| 3D-UNet | 0.482±0.124 | 0.639±0.127 | 0.775±0.130 | 0.561±0.137 |
| 3D-Swin UNETR | 0.451±0.098 | 0.615±0.100 | 0.718±0.087 | 0.549±0.120 |
| **MDL with the IC technique** |  |  |  |  |
| 3D-UNet | **0.564±0.092** | **0.716±0.081** | 0.812±0.087 | **0.652±0.103** |
| 3D-Swin UNETR | 0.515±0.104 | 0.673±0.098 | 0.722±0.117 | 0.641±0.103 |
